# IrO_2_-Decorated Titania Nanotubes as Oxygen Evolution Anodes

**DOI:** 10.3390/molecules30142921

**Published:** 2025-07-10

**Authors:** Aikaterini Touni, Effrosyni Mitrousi, Patricia Carvalho, Maria Nikopoulou, Eleni Pavlidou, Dimitra A. Lambropoulou, Sotiris Sotiropoulos

**Affiliations:** 1 Department of Chemistry, Aristotle University of Thessaloniki, 54124 Thessaloniki, Greece; katerinatouni@gmail.com (A.T.); dlambro@chem.auth.gr (D.A.L.); 2 SINTEF Industry, Forskningsveien 1, NO-0373 Oslo, Norway; patricia.carvalho@sintef.no; 3 Department of Geology, Aristotle University of Thessaloniki, 54124 Thessaloniki, Greece; marthonik@geo.auth.gr; 4 Department of Physics, Aristotle University of Thessaloniki, 54124 Thessaloniki, Greece; elpavlid@auth.gr

**Keywords:** dimensionally stable anodes, acid water electrolysis, black titanium dioxide, iridium oxide, titanium dioxide nanotubes, galvanic replacement

## Abstract

In this work, we have used both plain titania nanotubes, TNTs, and their reduced black analogues, bTNTs, that bear metallic conductivity (prepared by solid state reaction of TNTs with CaH_2_ at 500 °C for 2 h), as catalyst supports for the oxygen evolution reaction (OER). Ir was subsequently been deposited on them by the galvanic replacement of electrodeposited Ni by Ir(IV) chloro-complexes; this was followed by Ir electrochemical anodization to IrO_2_. By carrying out the preparation of the TNTs in either two or one anodization steps, we were able to produce close-packed or open-structure nanotubes, respectively. In the former case, larger than 100 nm Ir aggregates were finally formed on the top face of the nanotubes (leading to partial or full surface coverage); in the latter case, Ir nanoparticles smaller than 100 nm were obtained, with some of them located inside the pores of the nanotubes, which retained a porous surface structure. The electrocatalytic activity of IrO_2_ supported on open-structure bTNTs towards OER is superior to that supported on close-packed bTNTs and TNTs, and its performance is comparable or better than that of similar electrodes reported in the literature (overpotential of *η* = 240 mV at 10 mA cm^−2^; current density of 70 mA cm^−2^ and mass specific current density of 258 mA mg_Ir_^−1^ at *η* = 300 mV). Furthermore, these electrodes demonstrated good medium-term stability, maintaining stable performance for 72 h at 10 mA cm^−2^ in acid.

## 1. Introduction

Ti-supported electrodes find applications in many industrial processes, most notably as dimensionally stable anodes (DSAs) in the chlor-alkali, electroplating and electrowinning industries [1,2,3] and as gas diffusion layer electrodes (GDLEs) in polymer electrolyte fuel cells (PEMFCs) and electrolyzers (PEMECs) [4,5,6,7]. When the anode reaction of interest is that of oxygen evolution (OER), IrO_2_ is the preferred catalyst (especially under acidic conditions) due to the typically slow kinetics and high overpotential associated with the OER [8,9,10] resulting in the need for efficient and stable electrode materials. Minimizing the Ir catalyst loading is crucial given its scarcity and high price; this can be achieved by preparing very thin continuous films or highly dispersed IrO_2_ particles over the Ti-based substrate [11,12,13,14,15]. In the latter case, to ensure electrode stability at high anodic potentials and/or aggressive conditions, there is a need for the presence of protective Ti oxides at uncovered locations (e.g., by thermal annealing) or the use of a TiO_2_ substrate itself [16,17,18,19,20]; these strategies, however, may induce particle agglomeration and/or increased ohmic losses due to thick TiO_2_ layer formation.

Depending on the fabrication route, titanium dioxide can exist in various morphologies, such as nanoparticles [21,22,23], nanowires [24,25], nanofibers [26,27], and nanosheets [28,29], each exhibiting distinct properties and offering a variety of potential applications. Among them, titania nanotubes (TNTs) have attracted considerable attention over the years owing to their high surface area and easily tunable geometrical parameters (nanotube length, pore size, tube orientation) [30,31,32]. In addition, electrochemical anodization of a Ti foil (the fabrication method most commonly used to produce nanotube structures), offers simplicity and low cost regarding the materials and instrumentation it requires. By adjusting the anodization parameters, such as duration, applied voltage, temperature and electrolyte composition, different nanotube morphologies can be obtained according to the preferred application [31,33,34]. Therefore, TNTs are ideal candidates to be used as OER catalyst supports, since they combine the inherent stability of titania oxides at high anodic potential values with a high surface area for the dispersion of IrO_2_ and an open structure for the evolved oxygen gas to escape.

One major drawback of titania nanotubes (and TiO_2_ in general) is the fact that they do not possess sufficient electronic conductivity and for that reason they should either be densely decorated by the conducting electrocatalyst or rendered themselves conducting. The conductivity of TiO_2_ can be greatly impacted by the presence of defect states (Ti^3+^ interstitials and $V_{O}^{\cdot \cdot }$ oxygen vacancies) in the crystal lattice or by the incorporation of different dopants to modify its band structure [35,36]. The former can be achieved by employing methods such as hydrogenation (annealing in a hydrogen rich atmosphere [37,38,39,40] or via hydrogen plasma treatment [41,42]), chemical reduction (via the reaction with strongly reducing reagents such as NaBH_4_ [43,44,45] and CaH_2_ [46,47]) or electrochemical reduction (by applying a sufficiently negative potential in buffered solutions) [48,49,50,51].

Most of the research studies reported in the literature focus on the photoelectrochemical applications of titania nanotubes, while few have explored their application as a support material for platinum group metal (PGM) catalysts to promote the oxygen evolution reaction (OER). Dehkordi et al. fabricated IrRuO_x_/TNT electrodes through a modified Adams’ fusion method with 80 % *w*/*w* loading (of an Ir:Ru ratio equal to 60:40 at%), which showed enhanced activity and stability towards the OER compared to IrRuO_x_ supported on TiO_2_ powder, due to the increased number of active sites and better catalyst distribution [52]. To lower the catalyst loading, it is essential to improve the electronic conductivity of the TNTs, which will facilitate the electron transfer from the catalyst surface to the substrate during the OER. With this in mind, Genova-Koleva et al. synthesized IrO_2_ and IrRuO_x_ catalysts supported on plain and Nb-doped TNTs with a 50 wt% catalyst loading, where the IrO_2_/Nb-TNTs stood out owing to the high surface area and enhanced conductivity of the support, due to the presence of Nb(IV) species, which also allowed for a better catalyst dispersion [53]. In another study, Lu et al. used hydrogenated TNTs as support and IrO_2_ was electrodeposited via potential cycling of the electrode substrate. The catalyst supported on the hydrogenated TNTs showed enhanced catalytic activity towards the OER compared to its plain semiconducting analogues, due to the higher deposition rate of IrO_2_ that resulted in higher surface-active area electrodes [54]. Other modifications of TNTs as supports for OER include titanium oxy-nitride [55,56]. Finally, apart from the chemical/electrochemical methods of Ir deposition onto TNTs one should also mention that of single atom photodeposition [57] and Atomic Layer Deposition (ALD) [58].

Similar catalyst structures (decorated with Pt or Ir) have also been investigated for the hydrogen evolution reaction (HER). More specifically, Lačnjevac et al. fabricated hydrogenated TNTs via cathodic reduction followed by galvanic deposition of metallic Ir from its IrCl_3_ precursor solution to obtain Ir/H-TNTs. The authors emphasize that the strong interaction between Ir and the conductive TNT support is pivotal in enhancing the catalytic efficiency of these Ir/H-TNTs for the HER [59]. More recently, Touni et al. prepared Pt/TNT electrodes for HER by spontaneous (galvanic) deposition of Pt onto freshly reduced TNTs by solid state reaction with CaH_2_ [60] that has rendered it black titania (b-TNT) [61]. To the best of our knowledge, although b-TNTs are widely used in photocatalysis they have not been employed as substrates for OER (presumably due to reservations regarding the possible re-oxidation of the partially reduced titania).

Our research group has focused on the production of PGM catalysts supported on various conducting substrates through the galvanic replacement/deposition method, which is driven by the difference in the standard potentials between a noble (e.g., Ir, Pt, Ru) and a less noble metal (e.g., Ni, Ti). Through galvanic replacement, a (mixed) core–shell or (mixed) core–skin structure is formed (depending on whether the substrate is in particulate or layer form), whereby the metal shell/skin is composed of the catalytic noble metal, and the core consists of a mixture of the noble metal with abundant, less expensive non-noble metals [15,62,63,64,65,66,67]. This process enhances the precious metal mass-specific activity, allowing for a reduction in PGM loading in electrocatalyst applications. Furthermore, the interaction between the shell/skin and the core components is expected to modify the catalytic properties, potentially improving overall performance [15,62,63,64,65,66,67].

To the best of our knowledge, galvanic replacement of a sacrificial metal by a noble metal has not yet been employed for the decoration of TNTs.

In this work, we investigate the feasibility of the galvanic replacement method on semiconducting or metallic-like black titania nanotubes, with either a close-packed or open structure, using Ni as a sacrificial layer and Ir as the depositing noble metal. The goal is to produce novel, efficient and stable IrO_x_ TNT-supported electrodes for use as OER anodes. SEM/EDS along with ICP-MS analysis was performed to assess the morphology and composition of the electrocatalysts. Electrochemical characterization was conducted by means of cyclic voltammetry (CV), near-steady state linear sweep voltammetry (LSV) and electrochemical impedance spectroscopy (EIS) measurements. Finally, the stability of the most active electrode towards the OER was investigated by means of constant current chronopotentiometry, while XPS analysis was conducted before and after the stability test to observe changes in its chemical composition. The novelty of this work has been two-fold: proving that galvanic replacement can work on TNTs as an alternative method for their decoration with noble metals and, also, that bTNTs can be used as stable substrates for OER catalysts.

## 2. Results

### 2.1. Microscopic (SEM) and Spectroscopic (EDS, ICP-MS, XPS) Analysis

**Figure 1:**
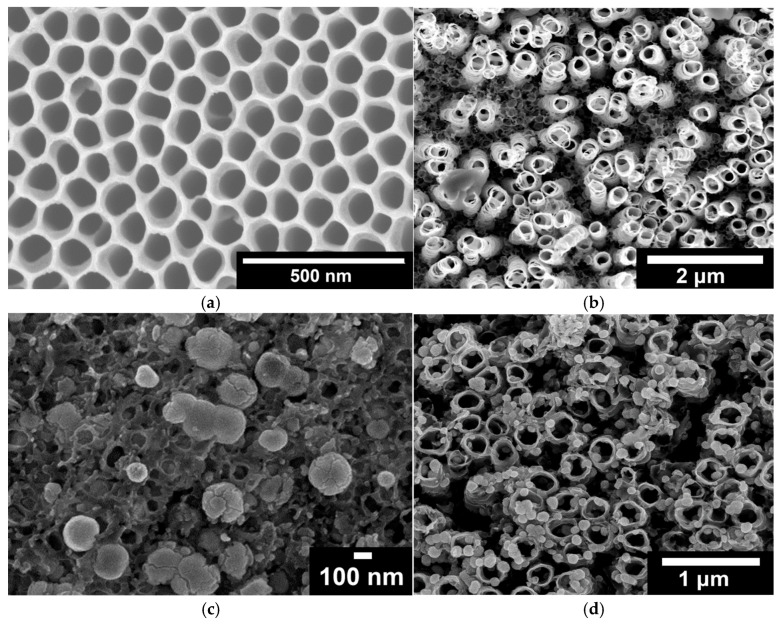
SEM micrographs of: (**a**) two-step/close-packed TNTs; (**b**) one-step/open-structure TNTs; (**c**) close-packed IrO_x_(Ni)/bTNTs and (**d**) open-structure IrO_x_(Ni)/bTNTs.

In the case of two-step bTNTs (Figure 1a), the nanotubes are close-packed, vertically oriented with a relatively smooth nanotube surface and distinct honeycomb-like morphology [46,68]. In contrast, the one-step bTNTs (Figure 1b) display a sparse arrangement of the nanotubes with an open structure. The difference in morphology may be attributed not only to the one- and two-step anodization processes but also to the use of a more water-rich anodization electrolyte in the case of open-structure nanotubes. In the two-step anodization process, the pre-formed pattern from the first step is utilized to grow highly ordered nanotubes, whereas in the one-step process, strict control over orientation is more challenging [69,70]. In addition, variations in the electrolyte composition further influence the structure. The water-rich electrolyte used to obtain open-structure TNTs results in increased solubility of the fluoride-rich layer of the nanotube walls, leading to a more open-structure with thinner walls [71,72,73]. Hence, the average nanotube diameter was 193 nm for the one-step/open structure (Figure 1b) and 92 nm for the two-step/close-packed (Figure 1a) TNTs.

**Figure 2:**
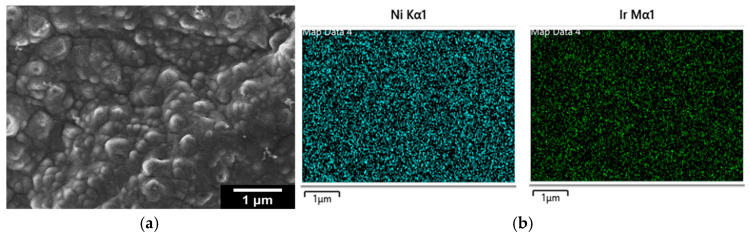
(**a**) SEM micrographs of close-packed IrO_x_(Ni)/TNTs; (**b**) the corresponding EDS mapping analysis.

Following electrodeposition of Ni and galvanic replacement by Ir, it appears that the surface of the close-packed bTNTs (Figure 1c) and TNTs (Figure 2) is covered by large (>100 nm) Ir-Ni particles/aggregates that are blocking most/all the pores of the nanotubes. EDS elemental analysis of these close-packed electrodes gave an Ir:Ni atomic ratio of ca. 6 for the bTNTs and 0.13 for the TNTs substrates, indicating a larger remaining Ni content in the latter case, in line with the larger quantity of initially electrodeposited Ni that was needed to grow Ni from the more conducting bottom of the TNTs to their top surface (see discussion in *Section 4.1.2* below and Figure S1). Also, in the case of the semiconducting-low electronic conductivity TNTs, Ir electron uptake/deposition is expected to occur only at conducting Ni sites (“protecting” them against further dissolution) and not on titania sites (as should be the case for electron-conducting bTNT sites, thereby leading to excessive Ni dissolution from neighboring uncovered Ni locations).

For the open-structure IrO_x_(Ni)/bTNTs (Figure 1d), Ir nanoparticles with an average diameter of 74 nm (56–93 nm) can be seen, some residing at the rim or the interior of the pores (where sacrificial Ni apparently could be pre-deposited) and no Ni was detected by EDS, indicating its complete dissolution/replacement. To confirm that all Ni has been dissolved even from the surface of the open-structure bTNTs (and therefore, that Ir has been deposited on conducting open-structure bTNT sites), XPS analysis was also carried out for the as-prepared sample and no Ni was detected, whereas Ir was detected at 4–7% atomic concentration depending on location.

**Figure 3:**
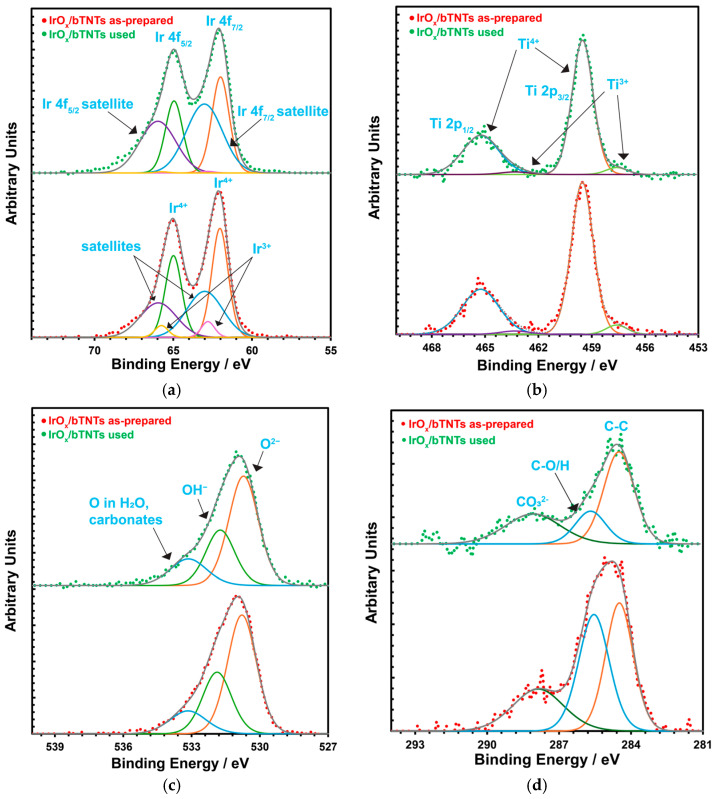
XPS spectra for the as-prepared (red dotted line) and used (green dotted line) for OER after 72 h IrO_x_/bTNTs electrode: (**a**) Ir 4f; (**b**) Ti 2p; (**c**) O 1s; and (**d**) C 1s spectra.

XPS measurements were also performed after the stability test of the open-structure IrO_x_/bTNT sample and no significant changes were observed in the surface chemical composition of the electrode. The corresponding XPS spectra for the as-prepared and used IrO_x_/bTNTs electrodes are shown in Figure 3. Figure 3a shows the Ir 4f spectrum, which consists of four main components. The 4f_7/2_ and 4f_5/2_ doublet corresponding to the main XPS 4f peaks for Ir^4+^ (IrO_2_) and their two associated satellite peaks (at a binding energy ~1 eV above the main peaks), which have their origin in the excitation of Fermi energy (E_F_) electrons to unoccupied state at ~1 eV above the E_F_ of IrO_2_. The main difference between the two samples is the satellite-to-main peak intensity ratio, which is higher for the “used” sample. This can indicate that IrO_2_ in the “used” sample has more available unoccupied states just above the Fermi level. Other possible interpretations could in principle be the presence of Ir^3+^, as its binding energy has been reported to be at a higher value than for Ir^4+^ [74] or the presence of hydroxides of Ir^4+^ [75]. However, and although there does not seem to be a consensus on the peak position and shape of the 4f peaks for Ir^3+^ [74,75], its presence is more likely in the as-prepared sample that has not been exposed to positive potentials/oxidative conditions, as also indicated by the need to include two additional peaks at 62.6 and 65.6 eV in that case (Figure 3a, bottom) to account for hydrated Ir^3+^ [75]. Figure 3b shows the Ti 2p spectrum, which corresponds to Ti^4+^ and Ti^3+^. The 4+/3+ peak intensity (area) ratio seems almost unchanged between the two samples. Figure 3c shows the O 1s spectrum, showing components attributed to oxides, hydroxides and compounds containing adsorbed oxygen species. No significant differences between the two samples are observed. Finally, Figure 3d gives the C1s spectrum, as this peak was used to slightly correct the energy scale after charge compensation by setting the C-C component to 284.5 eV. The “used” sample has less oxidized C, suggesting its removal during OER operation in the acidic environment.

Finally, Ir loading was determined as 0.3 mg cm^−2^ for the open-structure IrO_x_(Ni)/bTNTs by the ICP-MS technique (following electrode etching as described in *Section 4.3*). Since both XPS, EDS, and ICP-MS analysis revealed that Ni is not present in the open-structure bTNTs catalyst, it will henceforth be referred to as IrO_x_/bTNTs instead of IrO_x_(Ni)/bTNTs.

### 2.2. Electrochemical Characterization

#### 2.2.1. Surface Electrochemistry of the Catalysts

For the as-prepared Ir/Ni-, Ir-loaded electrodes were scanned multiple times in a 0.1 M HClO_4_ deaerated solution with a scan rate of 50 mV s^−1^, until stable voltammetry was recorded, in the potential range of

**Figure 4:**
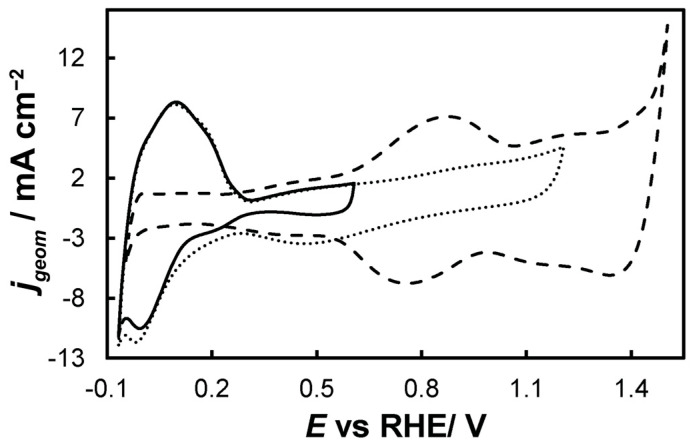
Stabilized cyclic voltammograms (obtained at 50 mV s^−1^, in deaerated 0.1 M HClO_4_) of the open-structure IrO_x_/bTNTs recorded in three potential windows, with an upper potential limit of +0.6 V_RHE_ (solid line), +1.2 V_RHE_ (dotted line), and +1.5 V_RHE_ (dashed line).

**Figure 5:**
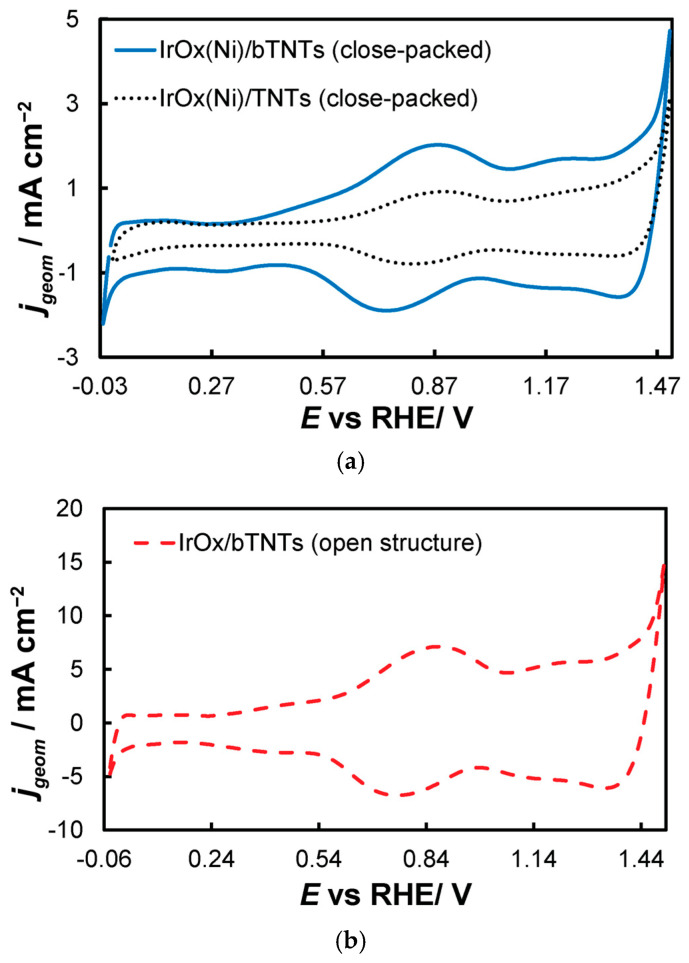
Stabilized cyclic voltammograms (obtained at 50 mV s^−1^, in deaerated 0.1 M HClO_4_) recorded in the potential window between hydrogen and oxygen evolution for the (**a**) close-packed IrO_x_(Ni)/bTNTs and TNTs and (**b**) open-structure IrO_x_/bTNTs. Current density, j_geom_, is per electrode substrate projected geometric area.

- 0.0 V_RHE_ to +0.6 V_RHE_, corresponding to the potential range between the onset of hydrogen evolution and the end of Ir double layer potential region [76,77], for the electrochemical dissolution of any surface uncovered/unreacted Ni and the formation of an Ir(Ni) mixed core–skin structure [65] in the samples that contain Ni, until only the peaks attributed to the adsorption and desorption of an under-potentially deposited hydrogen (UPD-H) layer on metallic Ir could be clearly recorded, as depicted indicatively in Figure 4 for the case of open-structure Ir/bTNTs. The electrochemical surface area (ECSA) of metallic Ir is related to the charge associated with the adsorption of a H monolayer on Ir; the former could be calculated from the voltammograms of Figure 4 (by integration of the anodic/H-desorption peak between −0.05 and 0.30 V) as 21.8, 49.4, and 239 cm^2^_Ir_ cm^−2^ for the close-packed Ir(Ni)/TNTs, the close-packed Ir(Ni)/bTNTs, and the open-structure Ir/bTNTs, respectively [15,64,78], in line with the increase in IrO_2_ coating roughness/particle dispersion depicted in Figure 2, Figure 1c, and Figure 1d, respectively, that correspond to these three different electrode types.
- 0.0 V_RHE_ to +1.2 V_RHE_, where the characteristic reversible Ir surface oxide/hydroxide peaks of the Ir (III) ⇌ Ir (IV) redox transformations are recorded at E_a_ = 0.85 V_RHE_ and E_c_ = 0.75 V_RHE_ [67,77], as can be seen in Figure 5 for all electrode types.
- 0.0 V_RHE_ to +1.5 V_RHE_, for the electrochemical anodization of metallic Ir to different oxidation states (IV, V) in order to form stable, porous 3D-IrO_x_, which also extends to the interior of the material [79,80,81]. In Figure 5, the electrochemistry due to anodically generated IrO_x_ appears above +1.0 _VRHE_ [67,82,83]. From the CVs, the charge corresponding to Ir oxides, which is representative of the electroactive surface area available for OER [84,85,86], was calculated (by integration of the anodic/IrO_x_ formation peaks between 0.30 and 1.40 V) as 16.6, 26, and 80 mC cm^−2^ for the close-packed IrO_x_(Ni)/TNT and IrO_x_(Ni)/bTNTs, as well as the open-structure IrO_x_/bTNTs, respectively (again, in line with coating roughness/particle dispersion shown in the SEM micrographs of Figure 1 and Figure 2). Taking into account the mass loading of Ir (see *Section 2.1* above), the mass specific electroactive area/oxide charge of the IrO_x_/bTNT electrode can thus be estimated as 267 C g_Ir_^−1^, a value that compares favorably with those of commercial IrO_2_ powder electrodes (100–200 C g_Ir_^−1^ [19,85]) and IrO_2_ supported on TiO_2_ powder electrodes (54–125 C g_Ir_^−1^ [19]).

#### 2.2.2. Oxygen Evolution Reaction

**Figure 6:**
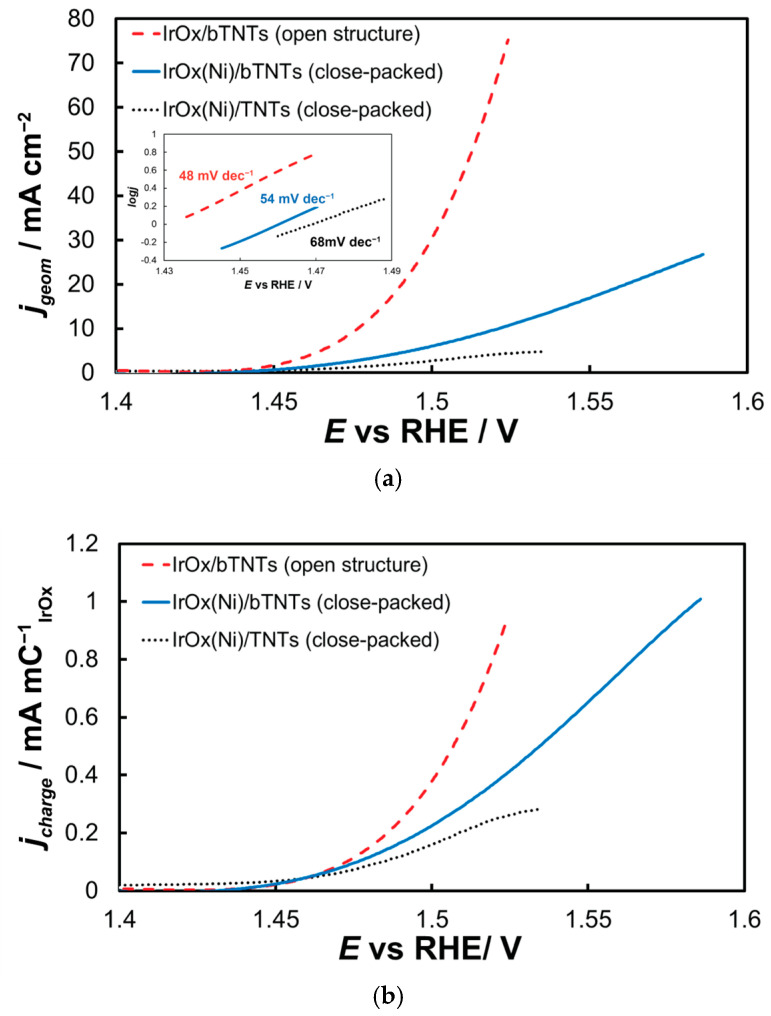
(**a**) Current density (per electrode substrate geometric area) vs. applied potential curves corrected for the uncompensated solution resistance (obtained at 5 mVs^−1^, in deaerated 0.1 M HClO_4_) and their corresponding Tafel plots (Inset). Current density, j_geom_, is per electrode substrate projected geometric area; (**b**) same as above but with current density, j_charge_, normalized per electroactive IrO_x_ charge.

The electrocatalytic electrodes were tested towards the OER by means of near-steady state experiments comprising LSV curves recorded at 5 mV s^−1^, from +1.2 to +1.7 V_RHE_. The current interrupt method was applied at +1.2, +1.3, +1.4, +1.5, and +1.6 V_RHE_ to estimate the uncompensated resistance and correct the applied potential for ohmic losses. The overall activity of IrO_x_ supported on untreated-semiconducting-low conductivity TNTs towards OER (Figure 6a) is inferior to that of IrO_x_ supported on reduced-conducting bTNTs, in line with its lower oxide electroactive surface area depicted in Figure 5 above and the continuous/non-particulate morphology of the corresponding deposit depicted in the SEM micrograph of Figure 2 above. Τhe IrO_2_ catalyst supported on the open-structure bTNTs exhibited the highest currents-activity towards OER. This can be attributed again to the higher electroactive surface area of IrO_2_ on these substrates (again, in accordance with its calculated IrO_x_ charge and its higher catalyst particle dispersion). Also, its open substrate morphology even after the introduction of the IrO_2_ nanoparticle catalyst, should prevent clogging of the nanotubes with generated O_2_ during the OER (which may be the case for the close-packed substrate electrodes). The Tafel slope for OER (*Inset* of Figure 6a) was estimated as 48, 54, and 68 mV dec^−1^ for the three electrode types, which is common for IrO_x_ supported electrodes and a mechanism based on a rds that entails the chemical activation of adsorbed OH species (produced after the first, one-electron transfer step from H_2_O to the anode electrode) [65,87,88].

Figure 6b presents data normalized for IrO_2_ quantity and dispersion as described by the charge related to its surface electrochemistry (by integration of the anodic part of Figure 5). Hence, current values reported in this way take into account differences in electroactive area between samples and are therefore representative of samples’ intrinsic activity (in the absence of any other parameters such as mass transfer limitations). Although the onset potential for OER appears to be similar for all electrodes studied, the superiority of the open-structure IrO_x_/bTNT electrode at higher overpotentials becomes apparent, pointing to the beneficial effect of the Ir-bTNT interactions (higher in the absence of remaining Ni on these samples-see Microscopic and Spectroscopic Characterization above) and/or of its open structure (see Figure 1d above) that limits extensive surface blockage by large oxygen bubbles.

#### 2.2.3. Electrochemical Impedance Spectroscopy (EIS)

**Figure 7:**
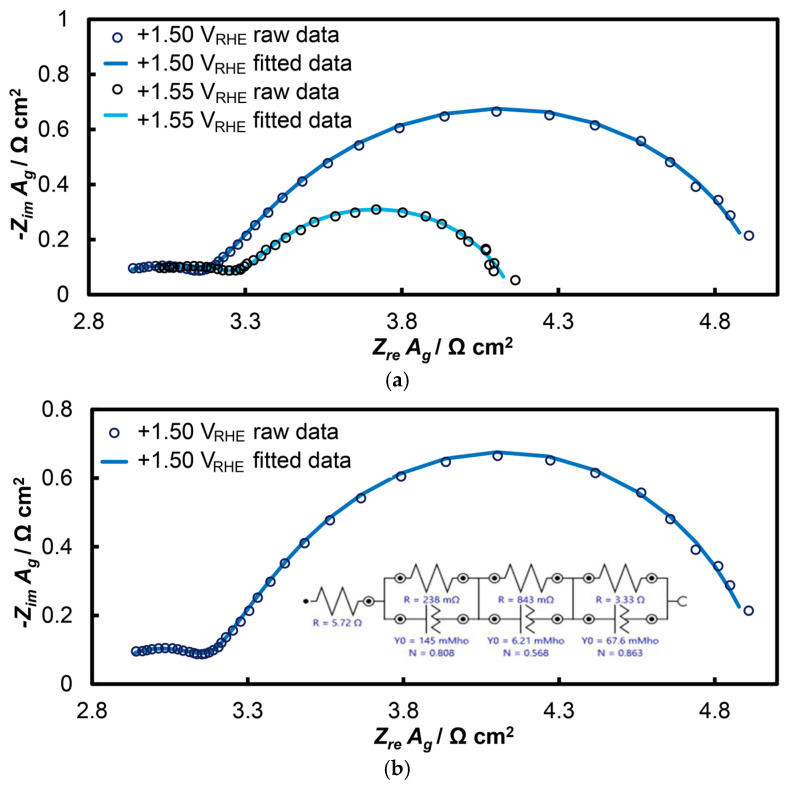
Nyquist plots of electrochemical impedance spectroscopy at the open-structure IrO_x_/bTNTs electrode: (**a**) at potentials of +1.50 and +1.55 V_RHE_ and (**b**) at the potential of E = +1.50 V_RHE_, as adapted to the equivalent electrical circuit *R_sol_*(*R_t_Q_t_*)(*R_p_Q_p_*)(*R_ct_Q_dl_*).

The response of the open-structure IrO_x_/bTNTs electrode is presented in Figure 7 (as Nyquist diagrams) at potentials of +1.50 and +1.55 V_RHE_ within the potential range of the OER. In these diagrams, two distinct semicircles can be observed, with the second (larger) semicircle showing slight deformation at higher frequencies, a phenomenon more pronounced in other similar systems [65,85]. This suggests the co-existence of three semicircles that can be modeled using the equivalent electrical circuit *R_sol_*(*R_t_Q_t_*)(*R_p_Q_p_*)(*R_ct_Q_dl_*). At very high oscillation frequencies, the solution resistance (*R_sol_*) was determined where the first semicircle intersects the real axis (*Z_re_*). Electrode porosity was also analyzed, with the first semicircle (modeled by *R_t_Q_t_*) attributed to the porosity of the IrO_x_/bTNTs system (as depicted by SEM), and the second semicircle (*R_p_Q_p_*) reflecting the nano-porosity of the 3D IrO_2_ oxides themselves. The pore resistance of iridium oxide is expected to be low, since the dispersed fine Ir particles cannot accommodate dense and thick IrO_x_ layers. At lower frequencies, the third circuit (*R_ct_Q_dl_*) corresponds to the OER, representing the electron transfer resistance to the IrO_2_ catalyst during OER and charging of the electroactive material double layer (i.e., associated with phenomena at the electroactive surface area/electrolyte solution interface where electron exchange during the OER occurs).

Table 1 below presents all the parameters of the equivalent electrical circuit *R_sol_*(*R_t_Q_t_*)(*R_p_Q_p_*)(*R_ct_Q_dl_*) fitted to the EIS data. The *R_ct_* is inversely related to the OER rate. When multiplied by the double layer capacitance, *C_dl_* (which is indicative of the actual surface of the electroactive material during the charge transfer of OER), it becomes the inverse of a time constant, representing in an inverse manner the intrinsic electrocatalytic activity of an electrode [85]. *Q_dl_* (the constant phase element, CPE, circuit component) was converted to *C_dl_* using the Mansfeld-Hsu equation [89,90] (Equation (1)):

(1) $$C_{dl}=Y_{dl}\;\omega^{{n_{dl}}^{-1}},\;\mathrm{where}\;\omega ={\left(\frac{1}{R_{ct}Y_{dl}}\right)}^{n_{dl}^{-1}}$$

#### 2.2.4. Stability Testing

**Figure 8:**
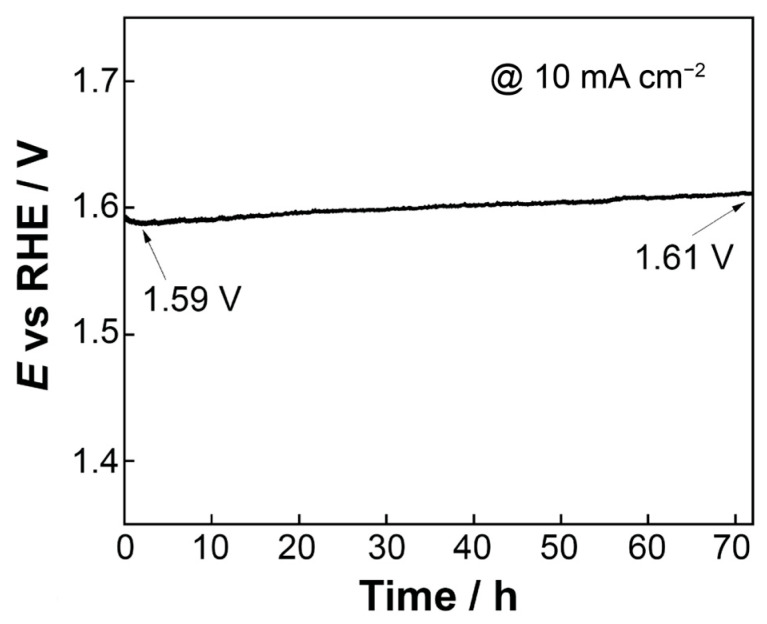
Potential vs. time curve (chronopotentiometry) at a constant current density of 10 mA cm^−2^ for 72 h in 0.1 M HClO_4_ electrolyte.

To assess the stability of the most electrochemically active electrode (open-structure IrO_x_/bTNTs) towards the OER, chronopotentiometry was employed and a current density equal to 10 mA cm^−2^ was applied for 72 h in 0.1 M HClO_4_ electrolyte. As can be seen from Figure 8, the electrode showed significant stability in the harsh acidic conditions during the OER and only a small shift in the potential equal to ca 20 mV was recorded.

**Figure 9:**
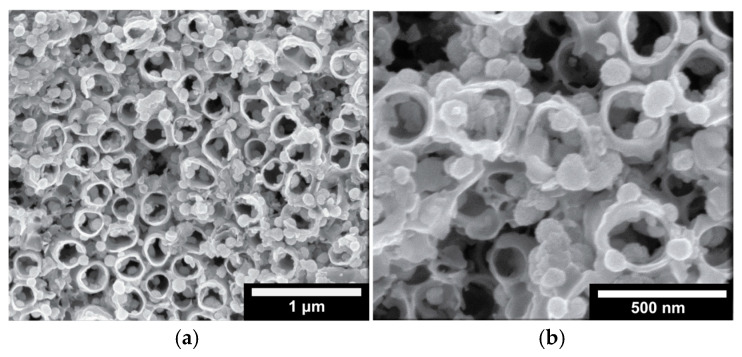
SEM micrographs of an open-structure IrO_x_(Ni)/bTNT after 72 h of continuous operation at 10 mA cm^−2^ in 0.1 M HClO_4_ electrolyte, at two different magnifications (**a**) and (**b**) as indicated by the scale bars (1 μm and 500 nm, respectively).

Furthermore, from the XPS measurements before and after the stability test (Figure 3) no significant changes in the chemical composition of the surface of the as-prepared and used electrode (IrO_x_/bTNTs) were observed, indicating the stability of both the substrate (bTNT) and the catalyst (IrO_x_) that ensures sustained conductivity and catalytic activity, respectively. This is also confirmed by the SEM micrographs of the sample, shown in Figure 9 below, whereby the morphology of the sample has not been changed when compared to that before the experiment, shown in Figure 1d. (Close inspection of the high resolution image of Figure 9b infers the partial filling of the nanotubes by Ir particles).

## 3. Discussion

### 3.1. Catalytic Electrode Morphology

As can be seen from the SEM micrograph of Figure 1d, it is the combination of good electronic conductivity (bTNT substrate) and open structure (one-step anodization) that ensures the formation of well-dispersed IrO_2_ nanoparticles on top of and inside the mouth of the pores of such samples. In all other cases (close-packed bTNTs and TNTs), a densely populated surface or a continuous IrO_2_ film is formed (Figure 1c and Figure 2a, respectively). In the former case, the open structure ensures solution contact with the entire substrate surface, hence facilitating the dissolution of pre-deposited Ni from all locations and the concurrent Ir deposition onto multiple conducting bTNT sites (during the galvanic replacement step). In the latter cases, the closed structure dictates Ni replacement preferentially on the surface of the substrate where it comes into contact with the Ir replacement solution. This interpretation is further supported by the fact that complete Ni dissolution was confirmed both by EDS, XPS, and ICP-MS experiments in the case of the open-structure bTNT substrates.

### 3.2. Catalytic Electrode Performance

As can be seen in Figure 6a OER catalytic activity per substrate (nominal-projected) geometric electrode area follows the trend: IrO_2_/bTNT > IrO_2_(Ni)/bTNT > IrO_2_(Ni)/TNT. This trend is due to a combination of surface area, intrinsic catalytic activity and mass transfer effects. Indeed, the same trend can be confirmed for catalyst dispersion/roughness by the corresponding SEM images of Figure 1 and Figure 2, as well as by the electroactive surface area itself, as estimated by the surface voltammetry shown in Figure 4 and Figure 5.

Normalizing for/removing the simple effect of surface area variation (Figure 6b) the trend remains the same and coincides with a decrease in Ni content/increase in direct IrO_2_-bTNT interactions. It seems, therefore, that these IrO_2_-bTNT interactions are more favorable to OER than those already reported for IrO_2_-Ni [63,65,67,86]. As stated in [60] and reported in references therein, bTNTs prepared via solid state reduction by CaH_2_ are characterized by the presence of neutral oxygen vacancies (with both the positive charge and electron polarons located at nearby Ti sites). These vacancies could act as sinks for O atoms of the IrO_x_ network destabilizing/activating the Ir-O-Ir bonds [86], a prerequisite of both the cation- and anion-type of mechanisms proposed for OER at IrO_2_ [91].

As far as mass transfer effects are concerned (expected to intervene at potentials higher than those of pure kinetic control, i.e., the ones within the potential range of the Tafel plots of the inset of Figure 6a, these are also expected to diminish as one moves from a film to a particulate and finally to a catalyst-decorated electrode, since oxygen bubbles formed during OER at various locations are less likely to coalesce and form a mass transfer barrier to reacting H_2_O. Indeed, the LSV curves corresponding to the IrO_2_(Ni)/TNT electrode (Figure 6) show clear signs of the onset of a current plateau at the more positive potential values studied, which is characteristic of mass-transfer limitations.

When it comes to comparison with external standards, the best performing electrode of this work (open-structure IrO_2_/bTNT) is estimated to show an overpotential of *η* = 240 mV at 10 mA cm^−2^ and current densities of 70 mA cm^−2^ and 258 mA mg_Ir_^−1^ at *η* = 300 mV. These values are comparable or better than similar electrodes reported in the literature (see Table 2).

As far as the EIS results are concerned, the existence of two semi-circles for the open-structure IrO_2_/bTNT electrodes (unlike film IrO_2_ or polymer-embedded IrO_2_ particle electrodes that give rise to a single, deformed semicircle [85]) is indicative for the existence of two types of porosity of different scales in our samples, one of the TNT-supported IrO_2_ and that of the porous 3D IrO_2_ itself that has not been previously reported. *R_ct_C_dl_* values (bearing units of a time constant and being representative-in a reverse manner-of intrinsic catalytic activity [85]) have been estimated to be in the 178–71 Ω mF (ms) range, which translates to TOF values of 5.62–14.08 s^−1^ for potentials in the +1.50–+1.55 V_RHE_ range. These TOF values are higher than the ones reported for anodically grown IrO_2_/Ir (0.4–0.286 s^−1^) [85] and IrO_x_(Ni)/GC electrodes prepared by galvanic replacement (0.496 s^−1^) [65], and comparable or better than those for IrO_x_(Ni)/Ti (11.48 s^−1^ [15]), confirming again the beneficial effect of the bTNT (and, to a smaller extent, of the TNT) substrate in enhancing the catalytic activity of IrO_2_ towards OER.

Finally, any reservations regarding the stability of the reduced bTNT support (that had to be used for best catalytic activity instead of TNTs) at the high anodic potentials of OER should be attenuated following the 72 h stable operation of these electrodes under OER in acidic conditions and the unchanged chemical states of both Ti and Ir, as confirmed by the XPS data of Figure 3.

## 4. Materials and Methods

### 4.1. Preparation of IrO_2_ Catalysts Supported on TNTs and bTNTs

#### 4.1.1. Preparation of Open-Structure and Close-Packed TNTs and bTNTs Substates

A Ti foil (0.25 mm thick, 99.5%, Thermo Fisher Scientific, Waltham, MA, USA) was cut into 1 cm × 1 cm squares and was sonicated for 10 min in acetone, ethanol, and doubly distilled (d.d.) H_2_O, to remove surface contaminants. After sonication, the Ti foil was left to dry in air before further use. The anodization took place in a one compartment cell, with Pt foil (2.5 cm × 1.3 cm) as the counter electrode/cathode, which was placed 2 cm away from the working electrode/anode (Ti foil). By applying 60 V through a DC power supply (Model DP60-15H, DSC Electronics, Bonn, Germany) in one anodization step for 120 min in glycerol solution (≥99.0%, Sigma Aldrich, Burlington, MA, USA) containing 0.5%wt NH_4_F (analytical grade, Sigma Aldrich), 10%wt d.d. H_2_O, we were able to obtain open-structure TNTs (ca 2 μm long) [73]. The close-packed TNTs were fabricated in two anodization steps by applying 60 V for 30 min + 90 min in ethylene glycol solution (analytical grade, Sigma Aldrich), containing 0.25wt% NH_4_F and 2wt% d.d. H_2_O [95] (resulting in 10 ± 2 μm long nanotubes). After the first anodization step, scotch tape was used to remove the nanotube film, and the patterned Ti foil was sonicated for 10 min in ethanol and left to dry before the second step. Throughout the experiment, the temperature was kept at 25 °C using a thermostat. To enhance the adhesion of the nanotubes to the Ti foil, the electrodes were kept in the anodization solution for 1 h [46]. The as-prepared TNT/Ti foils were annealed at 500 °C for 2 h in a muffle furnace with a heating rate of 1.7 °C min^−1^ to obtain TNTs with anatase structure. In the case of bTNTs, the substrate was annealed under the same heating conditions but in a sealed quartz ampoule in direct contact with CaH_2_ (CaH_2_ ≥ 97.0% powder, Sigma Aldrich), which resulted in the preparation of metallic-like conductive bTNTs [47,96].

#### 4.1.2. Preparation of IrO_x_(Ni)/TNTs and bTNTs

Initially, galvanostatic electrodeposition of Ni took place, in a one-unit cell in a Watt’s type of bath (30 g NiSO_4_•6H_2_O, 2.8 g NiCl_2_•6H_2_O, 4 g H_3_BO_3_ in 100 mL d.d. H_2_O), with Pt as the auxiliary electrode and saturated calomel electrode (SCE) as the reference electrode. The applied current density was equal to −5 mA cm^−2^ for close-packed TNTs, −3.7 mA cm^−2^ for close-packed bTNTs and −2 mA cm^−2^ for open-structure bTNTs, and it was selected based on the results of preliminary cyclic voltammetry (CV) scans to ensure Ni deposition occurred within the kinetic region/charge transfer control regime. The total charge density of the electrodeposited Ni was equal to 34–36 mC cm^−2^ for bTNTs and 29.2 C cm^−2^ for TNTs (with an expected 100% current efficiency for this type of Watts bath) and the temperature was held at 25 °C (bTNTs) and at 55 °C (TNTs) with constant magnetic stirring. The near thousand-fold higher charge density of electrodeposited Ni on the semiconducting TNTs was employed to achieve Ni penetration through the porous TNT substrate and down to its more conducting thin oxide/metal Ti base. In contrast to bTNTs, whereby nanotubes show good electronic conductivity along the nanotube walls, filling/partial filling of the pores of TNTs is essential to ensure electronic conductivity between the IrO_2_-decorated electrode surface and the Ti base current collector (with Ti at the bottom of the TNTs expected to possess higher conductivity due to a thinner surface oxide layer). Figure S1 of the Supplementary Material presents a SEM top-view micrograph of the bottom part of a Ni/TNT film (originally in contact with its Ti base), detached from a Ni/TNT/Ti electrode. It can be seen that deposited Ni at the bottom of TNTs follows the size and well-ordered pattern of the TNT matrix grown on the Ti basis, thus confirming that the filling of those TNTs starts from the TNT/Ti interface. This is in line with what has been reported for other annealed TNTs [97] and electrochemically treated amorphous TNTs [98].

Immediately following Ni deposition, the Ni/TNTs and Ni/bTNTs electrodes were immersed in a freshly prepared deaerated exchange solution (pH ≈ 3), consisting of 1 mM HCl + 1 mM K_2_IrCl_6_ (≥99.9% trace metal basis, Sigma Aldrich) for 15 min at 65 °C, for the galvanic replacement of Ni particles or layers by Ir. During galvanic replacement, metallic Ni is oxidized and dissolves as Ni^2+^ in the solution while Ir^4+^ is reduced to metallic Ir on the substrate surface (by electron uptake at Ni or conducting bTNT sites; Equation (2)), due to the positive difference in the standard reduction potentials of the Ir(IV)/Ir(III) (E^0^ = +0.86 V vs. SHE) and Ni(II)/Ni (E^0^ = –0.257 V vs. SHE) redox couples [62].

xNi + IrCl_6_^2–^ → Ir(x − 2)Ni + 2Ni^2+^ + 6Cl^–^ (2)

Finally, electrochemical anodization of metallic Ir was conducted by means of potential cycling with CV in 0.1 M HClO_4_ (70%, Merck, Darmstadt, Germany), in the potential range between H_2_/O_2_ evolution, to form porous IrO_x_ and dissolve any surface Ni not covered by Ir [15]. A three-electrode cell was used for this purpose, with Pt as the counter and SCE as the reference electrode.

### 4.2. Electrochemical Setup and Procedures

The electrochemical characterization of the as-prepared electrodes was conducted in a three-electrode cell, with Pt as the counter electrode and SCE as the reference electrode, which were separated from the working electrode by porous glass frits, to facilitate ionic conductivity in the bulk electrolyte solution. Cyclic and linear sweep voltammograms as well as EIS spectra were recorded with the help of an Autolab PGSTAT302N (Eco Chemie, Utrecht, The Netherlands) workstation, controlled via NOVA 1.11.2 software (Eco Chemie, Utrecht, The Netherlands). All electrochemical processes were carried out at room temperature.

CV was employed to investigate the surface electrochemistry of the catalysts, using consecutive scans in three potential ranges at a scan rate of 50 mV s^−1^ in 0.1 M HClO_4_ until stable voltammetry was recorded. The first range (−0.3 V to +0.3 V_SCE_) targeted the dissolution of unreacted Ni and the formation of an Ir(Ni) core–shell structure [65]. The electrode was transferred in a fresh deaerated 0.1 M HClO_4_ solution and scanned again. In the second range (−0.3 V to +0.9 V_SCE_), the formation of reversible Ir(III)/Ir(IV) oxides/hydroxides occurred, while in the third range (+0.3 V to +1.2 V_SCE_), anodization of Ir to higher oxidation states (Ir(IV) and Ir(V)) formed a stable, porous 3D-IrO_x_ structure [79,80,81].

Electrocatalytic activity towards the OER was evaluated using linear sweep voltammetry (LSV) at a potential sweep rate of 5 mV s^−1^ between +0.9 V and +1.4 V_SCE_. The current interrupt method was applied at 100 mV intervals in the +0.9 to +1.3 V_SCE_ potential range, during the potential scan, to estimate uncompensated resistance and correct the applied potential for ohmic losses. Chronopotentiometry was used to assess the stability of the electrodes for the OER by applying a constant current of 10 mA cm^−2^ for 72 h.

Finally, EIS measurements were carried out to verify the value of the solution resistance (R_s_) that was previously determined using the current interrupt technique during the LSV, and to calculate both the charge transfer resistance (R_ct_), characteristic of OER activity, and the double-layer capacitance (C_dl_), which is representative of the electroactive surface of the catalyst. EIS studies were conducted in the frequency range between 6 kHz and 0.1 Hz at DC potential values of +1.20 and +1.25 V_SCE_, recording the current response at an AC voltage amplitude of 10 mV. To find the equivalent circuit and determine values for the associated components, the NOVA 2.1 software was used.

The potential values reported are converted to the reversible hydrogen electrode (RHE) using the equation

E_RHE_ = E_SCE_ + 0.244 + (0.059 × pH) (3)

The equilibrium potential for the oxygen evolution reaction in 0.1 M HClO_4_ at room temperature is calculated as +0.927 V_SCE_.

### 4.3. Microscopic and Spectroscopic Characterization

The as-prepared TNTs and their modified bTNTs analogues were observed with Field-Emission Scanning Electron Microscopy (FESEM, JSM-7610F PLUS, JEOL Ltd., Akishima, Japan) supported by an Energy Dispersive X-ray Spectroscopy system (EDS, Oxford, Instruments Ltd., Oxford, UK), to obtain information about the morphology and the relative composition of the samples. Poorly conducting TNT samples were carbon-sputtered before measurements, while conducting bTNT samples were pictured as-prepared. In order to determine the quantity of deposited Ir supported on the best performing open-structure bTNTs, trace metal analysis was carried out with Inductively Coupled Plasma-Mass Spectroscopy (ICP-MS). The electrode was dissolved in boiling aqua regia (37% HCl, ChemLab; 65% HNO_3_, Merck) for 20 min and after reaching room temperature, the leachate was diluted in 2% *v/v* HNO_3_. The analysis was conducted by a Thermo Scientific iCAP Q ICP-MS, controlled via Q Tegra software (version 2.14.5122.158, Thermo Scientific). In addition, X-ray Photoelectron Spectroscopy (XPS) analysis was conducted to assess the surface atomic composition and the analysis was performed in a Thermo Scientific Thetaprobe X-ray Photoelectron Spectrometer, with a monochromated Al-Ka1 X-ray beam (*hν* = 1486.6 eV) and an analysis area of approximately 400 µm diameter.

## 5. Conclusions

- Electrodeposition of sacrificial Ni on conducting bTNTs and its subsequent galvanic replacement by Ir resulted (depending on substrate type) in Ir particles (<100 nm) for open-structure bTNTs or in larger aggregates for close-packed, bTNTs; in the case of open-structure bTNTs, these particles were highly dispersed (some residing inside the nanotubes), thus increasing the electroactive area while at the same time retaining an open electrode structure.
- For the semiconducting TNTs, an increase in the charge of electrodeposited Ni was necessary to rectify their original low electrical conductivity and resulting in the filling of the nanotubes for Ni deposits to act also as a current collector. The eventual formation of a continuous Ir(Ni) film on the surface following galvanic replacement, resulted in a significant decrease in the electroactive surface area.
- The open-structure IrO_x_(Ni)/bTNTs (more precisely, IrO_x_/bTNTs since no Ni has been detected after the galvanic replacement/metal exhange process), exhibited an enhanced activity towards the OER. This can be attributed to the higher surface area of the support, higher Ir dispersion and catalytic activity (due to IrO_2_-bTNT interactions) as well as less pore clogging during O_2_ evolution. An overpotential of η = 240 mV at 10 mA cm^−2^ and a mass-specific current density of 258 mA mg_Ir_^−1^ at η = 300 mV has been recorded, rendering them comparable or better than similar electrodes reported in the literature (in the 30–140 mA mg_Ir_^−1^ range at η = 300 mV and in the η = 240–360 mV range at 10 mA cm^−2^ [54,58,92,93]). Furthermore, the optimized electrodes, when tested for prolonged periods of time under OER conditions, were characterized by good short-term (72 h) stability.

## Figures and Tables

**Table 1:** Parameters of the equivalent circuit shown in Figure 7b at applied potential values of +1.50 and +1.55 V_RHE_ for the open-structure IrO_x_/bTNTs electrode.

Parameters		+1.50 V_RHE_	+1.55 V_RHE_
R_solution_ (R_sol_)	r_s_/Ω cm^2^	2.800	2.714
R_nanotubes_ (R_t_)	r_t_/Ω cm^2^	0.117	0.290
CPE_nanotubes_ (Q_t_)	y_t_/S s^n^ cm^−2^	0.296	0.001
	n_t_	0.808	0.635
R_pores_ (R_p_)	r_p_/Ω cm^2^	0.413	0.298
CPE_pores_ (Q_p_)	y_p_/S s^n^ cm^−2^	0.013	0.012
	n_p_	0.568	0.643
R_chargetransfer_ (R_ct_)	r_ct_/Ω cm^2^	1.632	0.851
CPE_double layer_ (Q_dl_)	y_dl_/S s^n^ cm^−2^	0.138	0.144
	n_dl_	0.863	0.792
χ^2^		0.00019 ^1^	0.00033 ^1^
C_dl_/mF cm^−2^		108.8	83.1
R_ct_C_dl_/Ω F (s)		0.178	0.071

^1^ The excellent fitting of the model to the raw data is confirmed by the very low *χ*^2^ values of the Kramers-Kronig test.

**Table 2:** Comparison of the area and mass specific activity towards OER of electrodes prepared in this work with similar electrodes reported in the literature.

Catalyst	Support	*η* @ 10 mA cm^−2^ (mV)	*J*_geom_ @ 300 mV (mA cm^−2^)	*J*_mass_ @ 300 mV (A g_Ir_^−1^)	Ref.
IrO_x_	bTNTs	240	70	258	This work
IrO_2_	TNTs	360	3.9	–	[92]
IrO_2_	Self-doped TNTs	–	–	116	[93]
IrO_2_	Hydrogenated TNTs	–	0.68	29.5	[54]
Ir	TNTs	240	23	143	[58]
Ir	TiO_x_N_y_ NTs	–	–	286	[94]

## Data Availability

The raw data supporting the conclusions of this article will be made available by the authors on request.

## Supplementary Materials

The following supporting information can be downloaded at: https://www.mdpi.com/article/10.3390/molecules30142921/s1, Figure S1: SEM top-view micrograph of the bottom part of an as-prepared Ni/TNT film peeled off the Ti support, Figure S2: SEM cross-sectional view micrograph of an Ir/Ni/TNT film, Figure S3: EDS spectrum of the Ir/Ni/TNT film depicted in Figure 2 [99].

## Abbreviations

The following abbreviations are used in this manuscript:

TNTs	Titania Nanotubes
bTNTS	Titania Black Nanotubes
CV	Cyclic Voltammogram/Voltammetry
LSV	Linear Sweep Voltammogram/Voltammetry
EIS	Electrochemical Impedance Spectroscopy
ICP-MS	Inductively Coupled Plasma Mass Spectrometry
SEM	Scanning Electron Microscopy
EDS	Energy Dispersive Spectroscopy
XPS	X-ray Photoelectron Spectroscopy
