# Supplementary material for: IrO2-Decorated Titania Nanotubes as Oxygen Evolution Anodes
Source: Molecules. 2025 Jul 10;30(14):2921. doi: 10.3390/molecules30142921 (PMC12300787; doi:10.3390/molecules30142921)
Supplement: Supplementary file 1 [file molecules-30-02921-s001.zip › molecules-3696690-supplementary.pdf]

Supporting Information

IrO_2-decorated Titania Nanotubes as Oxygen Evolution Anodes

Aikaterini Touni [1], Effrosyni Mitrousi [1,*], Patricia Carvalho [2] , Maria Nikopoulou [3], Eleni Pavlidou [4], Dimitra A. Lambropoulou [1] and Sotiris Sotiropoulos [1,*]

[1] Department of Chemistry, Aristotle University of Thessaloniki, 54124, Thessaloniki, Greece
[2] Department of Chemistry, Centre for Materials Science and Nanotechnology, University of Oslo, Gaustadalléen 21, NO-0349 Oslo, Norway
[3] SINTEF Industry, *Forskningsveien 1, NO-0373 Oslo, Norway*
[4] Department of Geology, Aristotle University of Thessaloniki, 54124, Thessaloniki, Greece
[5] Department of Physics, Aristotle University of Thessaloniki, 54124, Thessaloniki, Greece
* Correspondence: mitrouse@chem.auth.gr (E.M.); eczss@chem.auth.gr (S.S.)

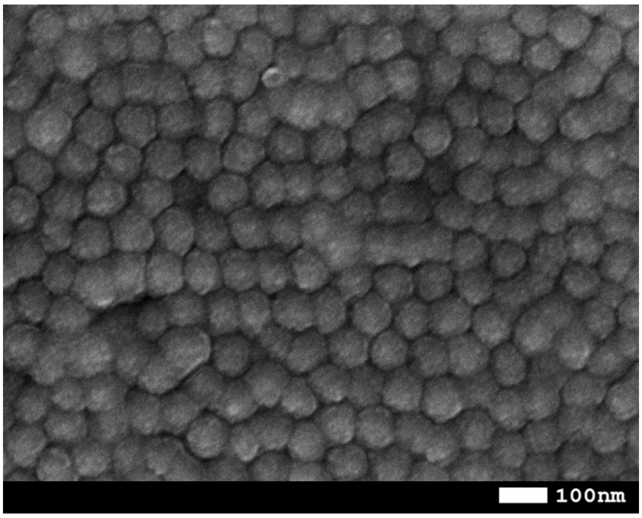

Figure S1 SEM top-view micrograph of the bottom part of an as-prepared Ni/TNT film peeled off the Ti support

In order to confirm that the filling of the semiconducting TNTs with Ni starts at the bottom of the TNT/Ti interface, a Ni/TNT/Ti electrode was prepared using identical conditions to the IrO_x(Ni)/TNTs electrode ($J_{el/dep}$ = -2 mA cm^{-2}, q_{Ni} = 29.2 C cm^{-2}). The as-prepared Ni/TNT film was peeled off the Ti support and its bottom view can be seen in Figure S1 above. The Ni deposit follows the well-ordered patterned structure of the close-packed TNT substrate with an average particle diameter of 90nm, which is in line with the pore nanotube diameter of the close-packed TNTs (see SEM micrograph of Figure 1a). This bottom-view of the Ni/TNTs is clearly distinguishable from its front view (Figure 2), where a dense structure persists after galvanic replacement, with the top surface appearing severely clogged. Note that similar SEM pictures and arguments for template-type metal deposition from the bottom of TNTs have appeared in the literature (see for example supplementary material of [1]).

The cross-sectional SEM micrograph of an Ir/Ni/TNT film shown in Figure S2 below infers the partial filling of the nanotubes by chains of metallic particles (seen as bright spherical features at locations). This is confirmed by EDS analysis of the same area that gave a 1.2% Ir - 55.3% Ni - 43.5% Ti atomic composition. (Note that similar morphologies for metal particles partially filling the pores of TNTs via electrodeposition appear in the literature-see for example [2].)

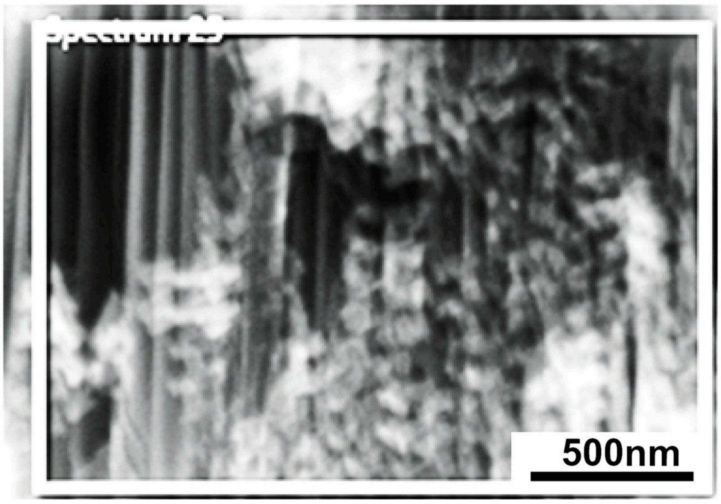

Figure S2 SEM cross-sectional view micrograph of an Ir/Ni/TNT film.

Finally, Figure S3 below presents the original data of the EDS spectrum obtained for the Ir/Ni/TNT film shown in Figure 2.

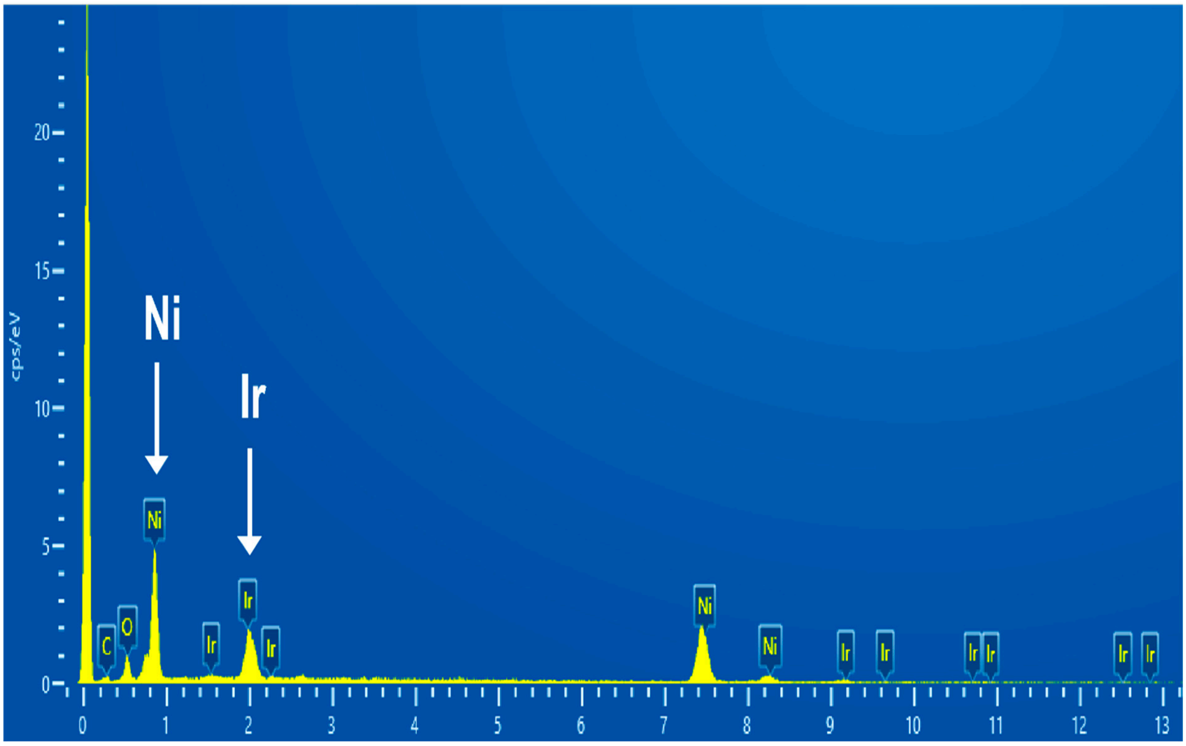

Figure S3 EDS spectrum of the Ir/Ni/TNT film depicted in Fig.2

References

1. Liang, F.; Zhang, J.; Zheng, L.; Tsang, C.K.; Li, H.; Shu, S.; Cheng, H.; Li, Y.Y. Selective Electrodeposition of Ni into the Intertubular Voids of Anodic TiO2 Nanotubes for Improved Photocatalytic Properties. *J Mater Res* **2013**, *28*, 405–410, doi:10.1557/jmr.2012.380.
2. Xu, B.; He, Y.; Zhang, Y.; Ma, Z.; Zhang, Y.; Song, W. In Situ Growth of Tunable Gold Nanoparticles by Titania Nanotubes Templated Electrodeposition for Improving Osteogenesis through Modulating Macrophages Polarization. *ACS Appl Mater Interfaces* **2022**, *14*, 50520–50533, doi:10.1021/acsami.2c13976.
